# Chargeability study of disinfectants and the optimization of design parameters of a handheld electrostatic disinfection device for small scale applications

**DOI:** 10.1371/journal.pone.0286740

**Published:** 2023-06-08

**Authors:** Aarti Chauhan, Manoj Kumar Patel, Manoj Kumar Nayak, Surender Singh Saini

**Affiliations:** 1 Academy of Scientific and Innovative Research (AcSIR), Ghaziabad, Uttar Pradesh, India; 2 Manufacturing Science and Instrumentation (MSI), CSIR–Central Scientific Instruments Organisation, Chandigarh, Chandigarh, India; 3 Materials Science and Sensor Applications (MSSA), CSIR–Central Scientific Instruments Organisation, Chandigarh, Chandigarh, India; 4 Indo Swiss Training Centre (ISTC), CSIR–Central Scientific Instruments Organisation, Chandigarh, Chandigarh, India; William & Mary, UNITED STATES

## Abstract

Apart from aerosols, contaminated surfaces with SARS-CoV-2 virus are the significant carriers of virus transmission. The disinfection and sanitization of the indoor and outdoor places are one among the powerful and effective strategies to avoid the surface-to-human transmission of SARS-CoV-2 (Severe Acute Respiratory Syndrome Coronavirus 2) through frequent touch and physical contact. Electrostatic spraying is one of the effective and efficient methods to apply the liquid-based sprays on surfaces to be disinfected or sanitized. This technique covers the directly exposed and obscured surfaces uniformly and reaches to hidden areas of the target. In this paper, the design and performance parameters of a motorized pressure-nozzle based handheld electrostatic disinfection device were optimized and the chargeability of ethanol (*C*_2_*H*_5_*OH*), formaldehyde (*CH*_2_*O*), glutaraldehyde (*C*_5_*H*_8_*O*_2_), hydrogen peroxide (*H*_2_*O*_2_), phenol (*C*_6_*H*_5_*OH*) and sodium hypochlorite (*NaClO*) has been critically investigated. The chargeability indicator for disinfectants was presented in terms of the charge-to-mass ratio. The significant value of the charge-to-mass ratio of 1.82 mC/kg was achieved at an applied voltage of 2.0 kV, the liquid flow rate and pressure of 28 ml/min and 5 MPa, respectively. The experimental results are well aligned to the proposed theoretical context.

## 1. Introduction

Following COVID-19, the environment has become an underestimated source of viral transmission and pathogenic infections. During the first few months of the outbreak of coronavirus disease, there was no evidence that the virus (SARS CoV-19) causing this disease is spreading through contaminated surfaces [1–3]. It was believed that the virus disseminated from person-to-person through physical contact or aerosol transmission from the infected person to the healthy individual through cough or sneeze [4]. Research findings suggest that besides virus- laden aerosol transmission, surface contamination in the vicinity of COVID-19 patients present in healthcare settings lead to the spread of virus through physical touch of infected surfaces such as hospital bed, walls, door handle, table, chairs etc. Also, the community shared settings such as markets, parks, transportation, educational institutes have become are the hotspots for the spread of viruses and pathogens [5–8]. Studies of the persistence time of virus on inanimate surfaces vary from hours to days [9, 10]. Therefore, environmental cleaning and disinfection is needed to stop the spread of the viruses [11]. Cleaning reduces visible dust on surfaces, however, it is inefficient for killing pathogens and viruses [12]. Moreover, manual cleaning is a time consuming and inefficient method of reducing disease causing microorganisms from hidden or curved parts of target surfaces [13].

Disinfection using spray technology is a feasible method to achieve the decontamination of indoor and outdoor public places and inanimate surfaces. Various spraying methods include conventional manual sprayers, fogging, fumigation and electrostatically charged sprays for surface disinfection [14, 15]. The conventional manual sprayers do not provide sufficient pressure to atomise the stream of liquid into droplets which is required for effective spraying [16, 17]. Whereas, electrostatic spraying technique emits charged spray droplets that stick to object surface thoroughly and eliminates the existing viruses and bacteria, effectively [18]. Whereas, fogging and fumigation methods outpour fog or mist of chemical disinfectants in the closed room or open environment that remains in that space for a long time and sometimes require de-fogging [19, 20]. Other prevalent technique for disinfection is UV-C that emits radiations in the range of 254 nm is said to be effective in reducing microbial growth, but suffers from the drawback of penetration to the curved or hidden surfaces. Moreover, UV-C cannot be employed in a space full of people with concern to the emission of radiation [21–23]. As per the literature surveyed, air-assisted electrostatic sprayers are more prevalent in the field of agriculture pesticide spray [24, 25]. Some of these are even used for disinfecting vegetation and crops against food pathogens [26]. Other disinfection sprayer available is about backpack sprayer type that do not achieve the required pressure and droplet size for effective surface disinfection [27]. Air-induced air-assisted electrostatic sprayers find widespread use as surface disinfectant. In these, the compressed air coverts the liquid stream into fine droplets in the size range of microns which are further electrostatically charged at the outlet [28]. The charged droplets after achieving a minimum of 1 mC/kg charge-to-mass ratio is capable of creating the wraparound effect on the target object [29, 30]. The charge-to-mass ratio is an important factor in electrostatic sprayers for efficient mass-transmission of spray onto the surface [31]. It is dependent on several design parameters vis-a-vis applied high voltage, applied air pressure, nozzle type and orifice diameter, flow rate, electrode shape and size, charging electrode position, electrode material and target distance [32–34]. Other than the design parameters, the performance parameters such as droplet size and its distribution, uniformity coefficient, spray deposition, horizontal and vertical spray losses and bio-efficacy are also needs to be optimized for effective spraying.

Various types of spraying devices are available based on the two fundamental design concepts and working principles: 1) Air-induced air-assisted based electrostatic disinfection device, 2) Hand-pressure based electrostatic disinfection device [35, 36]. In air-induced air-assisted electrostatic spraying device, the air supply and liquid supply meets at atomisation zone and converts the liquid stream into the fine droplets. The compressed air at high pressure mixes with the stream of liquid at the outlet of thin cavity that precedes the opening of the nozzle and divides it into numerous small-sized droplets. Numerous researchers have worked on air-assisted electrostatic spraying applied for various societal and industrial applications such as agriculture, environment, food safety, disinfection, painting, thin film deposition and powder coating [37–42]. In the case of hand-pressure based electrostatic spraying device, the pressure provided by hand pumping is not high enough to scatter the water stream into fine-sized droplets which is required for effective spraying [43]. In air-induced air-assisted electrostatic spraying, the compressor assembly makes the system bulky and costly to be afforded by household setups and small businesses and the hand-pressure based electrostatic disinfection device is not capable of generating the required pressure. To overcome this bottleneck, a third concept of motorized electrostatic spraying has been presented in this paper. In this method, the pressurised liquid is supplied to the nozzle by liquid pump. The liquid pump generates sufficient pressure to convert the stream of liquid into desired droplet size. The aim of our study is to replace the air-assisted electrostatic system with a motorized electrostatic disinfection device that will make it portable and cost effective.

To achieve this, the design parameters vis-à-vis applied high voltage, applied liquid pressure, liquid flow rate, charging electrode position, shape and size were optimized to achieve the maximum charge-to-mass ratio for efficient performance of motorized handheld electrostatic disinfection device. Higher is the charge-to-mass ratio, better is the performance in terms of deposition efficiency and wraparound effect [44–47]. However, charge-to-mass ratio cannot be increased infinitesimally at the cost of a particular parameter. Therefore, there is a trade-off among the various parameters and a critical assessment has been carried out on the chargeability of various disinfectants for effective and efficient disinfection and sanitization of inanimate surfaces.

## 2. Material and methods

### 2.1 Design and development of a pressure-nozzle based motorized handheld electrostatic disinfection device

The design and development of a handheld electrostatic disinfection device majorly consist of 1) Mechanical design and fabrication, 2) Electronic design and fabrication and, 3) the performance evaluation of the designed and developed electrostatic disinfection device on various disinfectants for chargeability studies. The developed handheld electrostatic disinfection device consists of a liquid pump, axial fan, pressure-based nozzle, disinfectant charging mechanism of nozzle and charging electrode, high voltage power supply for the charging of disinfectant and a switch for the switching (ON-OFF) of the high voltage power supply to charging electrode.

**Fig 1:**
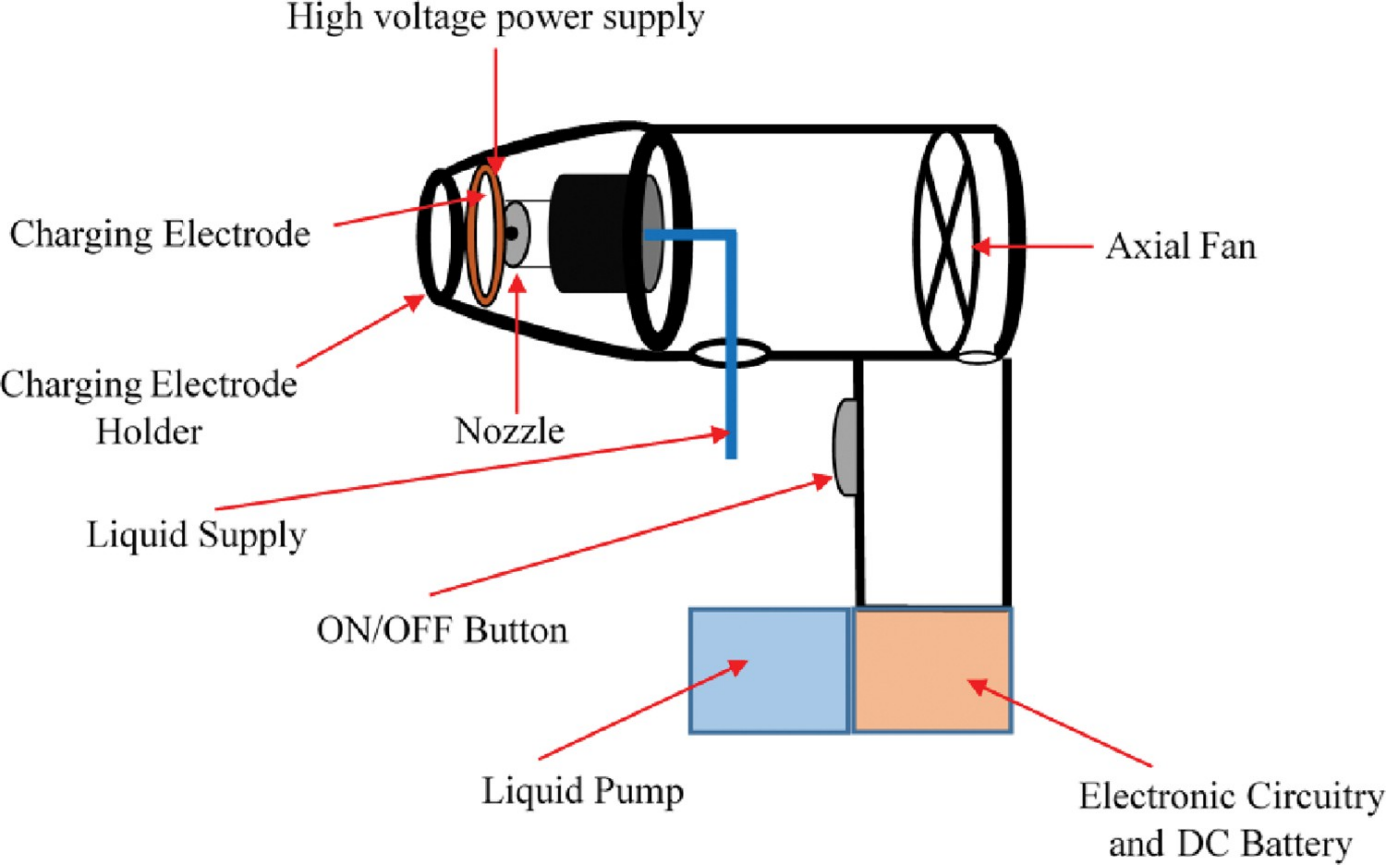
Schematic diagram of pressure-nozzle based handheld electrostatic disinfection device for surface decontamination.

The position of charging electrode from the nozzle tip, axial fan specifications, selection of liquid pump and the design of application specific and customised high voltage power supply are the critical components for the design and development of a handheld electrostatic disinfection device. The disinfectant is supplied to nozzle by a liquid pump through a liquid pipe connected to disinfectant tank. The high voltage is applied to charging electrode through a designed application specific high voltage power supply unit which is placed in the separate portion the sprayer [48]. The axial fan positioned at the rear end of the sprayer assists in the spread of liquid spray to a longer distance. The air flow provided by the axial fan also avoids the wetting of charging electrode. The push button acts as an ON/OFF switch for the switching of power supply to charging electrode as shown in Fig 1.

### 2.2 Physical properties of disinfectants

Physical and chemical properties are the important factors to define the chargeability of disinfectants. It is very important to note that, the developed electrostatic disinfection device should be designed in such a way that it must cover entire possible resistivity range of disinfectant without the compromise in performance of spraying device. Surface tension forces also play an important role in atomization process. The formation of droplets is difficult with liquids having higher surface tension. Atomization of droplets takes place when the inward acting surface tension force is less that the external electrostatic force. According to the Rayleigh charge limit of the droplet as expressed in Eq 1, both density and surface tension are the parameters that influence the charge-to-mass ratio as indicated in the Eq 4.

$$Qmax=8\pi \surd Ɛo\surd \sigma d^{3/2}$$ (1)

where *Qmax* is the Rayleigh charge limit, *Ɛ*_*0*_ is the free space permittivity, *σ* is the surface tension of droplet (*N/m*) and *d* is the diameter of droplet (*m*) [33, 34].

For the design and development of a handheld electrostatic disinfection device, the appropriate material has been used for the fabrication of various components. The selection of material was based on the merit of the capability to withstand the high voltage to avoid the electrical breakdown and the chemical inertness to avoid the corrosion and wear and tear. For the experiments, the high voltage was supplied by Ultravolt One Channel (Model: HV-RACK-1-250-00287, 20 kV, 1.5 mA, 30 W) and the pressure was varied using the customized liquid pump that has the facility to give out liquid at different pressures. The spray current was measured by using an electrometer (Make: Keithley, Model: 6514) to calculate the charge-to-mass ratio. To measure the charge-to-mass ratio, the older technique of the Faraday Cage method was used which was designed and developed in the laboratory. Two types of nozzles i.e., 0.15 mm and 0.20 mm were used for the experiments. To study the effect of shape and size of the charging electrode, 15 different sized charging electrodes were used.

The Laser Diffraction Sensor (Make: Sympatec, Model: HELOS-VARIO/KR) has been used for the measurement of the droplet size and its distribution generated by the electrostatic disinfection device [49]. The Laser Diffraction Sensor, based on the principle of dynamic light scattering, gives various parameters such as $x_{10,3},x_{16,3},x_{50,3},x_{84,3},x_{90,3},x_{99,3}$, C_opt_, *etc*., which are explained in the results and discussion section 4.6. Initially, the experiments were carried out using tap water for the optimization of parameters. For the optimization of parameters, one of the parameters was kept constant and other parameters were varied to see the effect of that particular parameter.

For the study of the chargeability, various chemical disinfectants such as ethanol (75%), formaldehyde (1%), glutaraldehyde (1%), hydrogen peroxide (6%), phenol (2%) and sodium hypochlorite (6%) are considered [50]. The physical properties such as conductivity, surface tension, density, viscosity and pH has been considered in the study, which greatly influence the charge-to-mass ratio and their usage as disinfectants were also studied [51, 52]. The physical parameters were measured using the instruments available in lab setup- WTW InoLab® Multi 9620 IDS for pH and conductivity, Surface tensiometer, KYOWA, DY-300 for surface tension and density and Modular Compact Rheometer 302e from Anton-Paar for measuring viscosity. The physical properties of various disinfectants are listed in Table 1.

**Table 1:** Physical properties of various disinfectants for the chargeability studies and suitability for electrostatic spraying.

Name of the disinfectant	pH	Conductivity (S/m)	Density (g/cm^3^)	Viscosity (mPa.s)	Surface tension (mN/m)
**Ethanol (75%)**	6.2	1.5×10^−4^	0.9176	2.1960	8.44
**Phenol (2%)**	5.4	1.6×10^−4^	1.0006	1.4218	34.10
**Hydrogen peroxide (6%)**	3.3	1.66×10^−2^	1.0269	0.9785	32.00
**Glutaraldehyde (1%)**	4.2	3.6×10^−3^	0.9906	0.8526	29.61
**Formaldehyde (1%)**	3.5	5×10^−3^	1.0230	0.7739	33.47
**Sodium hypochlorite (6%)**	12	1.8×10^−2^	1.1590	3.0878	36.53
**Tap water**	7.2	2.88×10^−2^	0.9966	1.7896	23.97

## 3. Experimental

The experiments have been carried out to optimise the design and performance parameters in order to develop a handheld electrostatic disinfection device. In this study, the charge-to-mass ratio has been considered as dependent variable which was calculated with respect to independent variables such as applied high voltage, liquid pressure, liquid flow rate, charging electrode position, charging electrode size and shape, charging electrode material, load current, spray coverage and resistivity range of the liquid to be sprayed. To carry out experiments for measuring the charge-to-mass ratio and the size of the droplet, tap water was used. The observed relative humidity and temperature during the experiments were 50 ± 5% and 290 ± 2K, respectively. The physical properties of the tap water used for the experiments are specified in Table 1.

### 3.1 Design of experimental set-up

We had taken round copper charging electrodes that varied in ring diameter and inner diameter. Ring diameter was chosen as 3 mm, 4 mm, 5 mm- each of which had an internal diameter of 11 mm, 12 mm, 13 mm, 14 mm and 15 mm making a total of 15 electrodes. Experiments have been carried out with each charging electrode by varying the applied voltage from 0 V to 2 kV. The distance between the electrode and nozzle was varied from 0 to 4 mm and the applied pressure was in the range of 4.5 MPa to 8 MPa.

**Fig 2:**
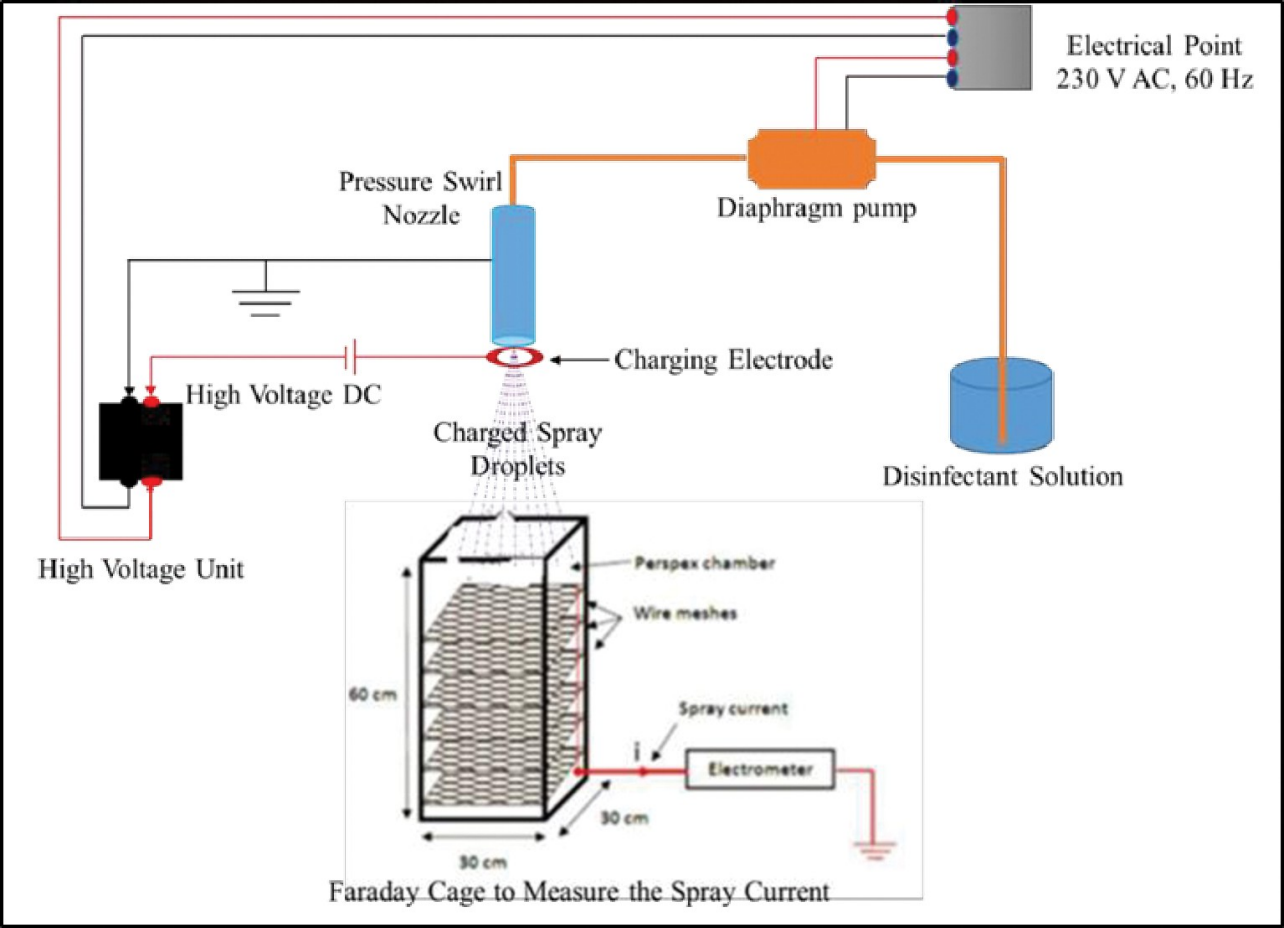
Pictorial representation of experimental setup for the optimization of parameters of the motorized handheld electrostatic disinfection device.

The variable high voltage is applied to the electrode and the water is pumped at a certain pressure by the motor that will emit out of the nozzle as a stream of tiny droplets. These droplets upon passing through the high voltage (HV) electrode will induce charge and fall on the Faraday cage which is further connected to the electrometer to give the value of spray current. The experimental setup is shown in Fig 2. The readings for each of the experiments were noted and corresponding graphs were plotted. After obtaining the maximum charge-to-mass ratio for a certain set of parameters, i.e., nozzle and electrode size, applied voltage and pressure, distance between nozzle and electrode- these were used for measuring the charge-to-mass ratio of chemical disinfectant solutions.

### 3.2 Charge-to-mass evaluation

For measuring charge-to-mass ratio, an application based Faraday Cage (fabricated at CSIR-CSIO) was used which was further connected to the earth potential via a digital multimeter (Brand: Keithley, Model: 6514) as shown in Fig 2. The charged droplets upon falling on the Faraday cage transfers the charge to the earth causing an electrical current which was measured by digital multimeter (electrometer) termed as ‘spray current’ [53]. Then this spray current was divided by the mass flow rate of spraying liquid to determine the charge-to-mass ratio. The charge-to-mass ratio was calculated from the relation:

$$Charge‐to‐mass\;ratio=i_{s}/Q_{m}\;mC/kg$$ (2)

**Fig 3:**
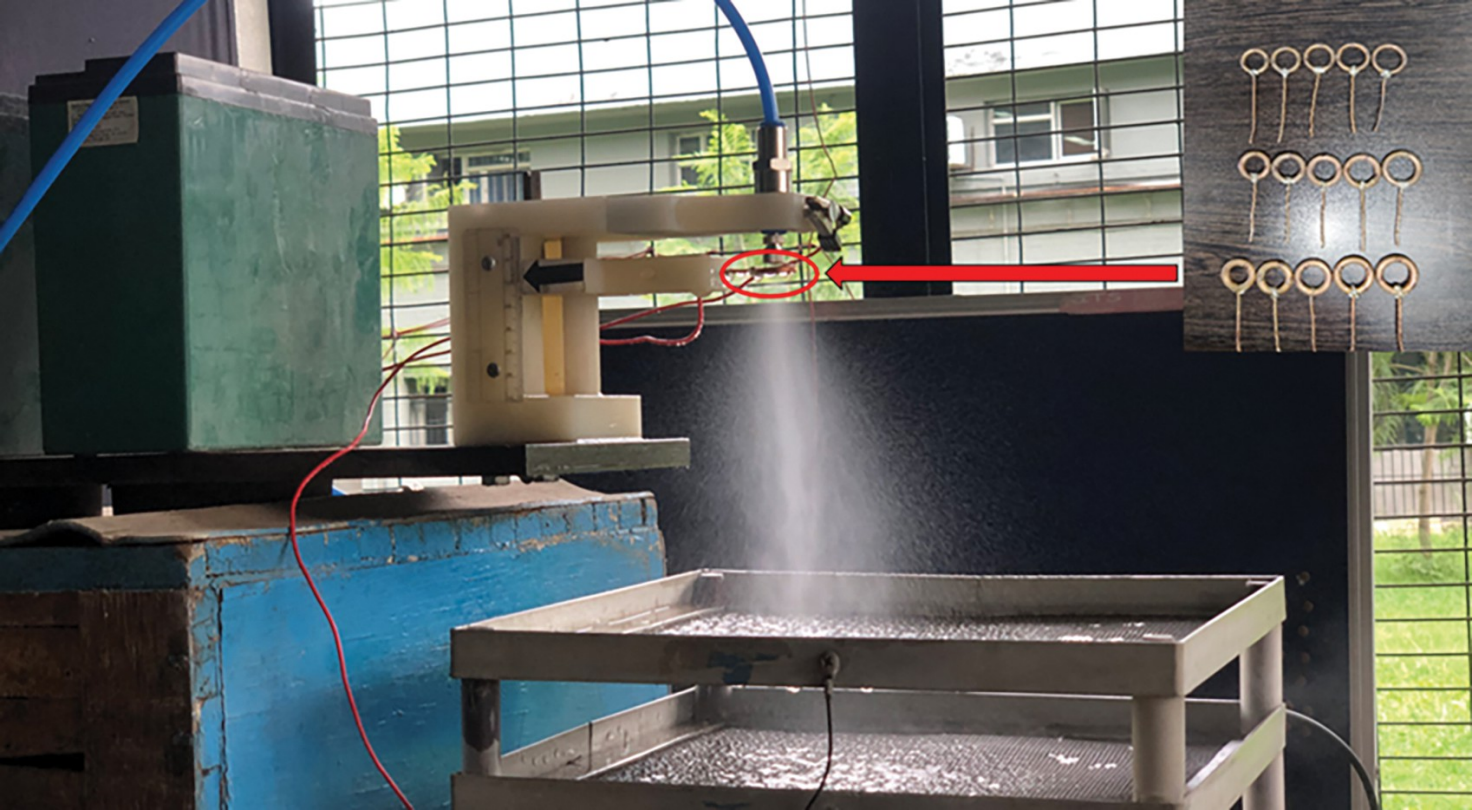
Experimental set-up of electrostatic disinfectant charging and charge-to-mass evaluation (inset: charging electrodes of varying inner and outer diameter for the charging of disinfectants).

Where *i*_*s*_ is the measured spray current (A) and *Q*_*m*_ is the mass flow rate of liquid (*kgs*^−1^). The prototype of the experimental setup is shown in Fig 3.

## 4. Results and discussion

### 4.1 Optimization of charge-to-mass ratio with varying position of charging electrode from the nozzle tip

**Fig 4:**
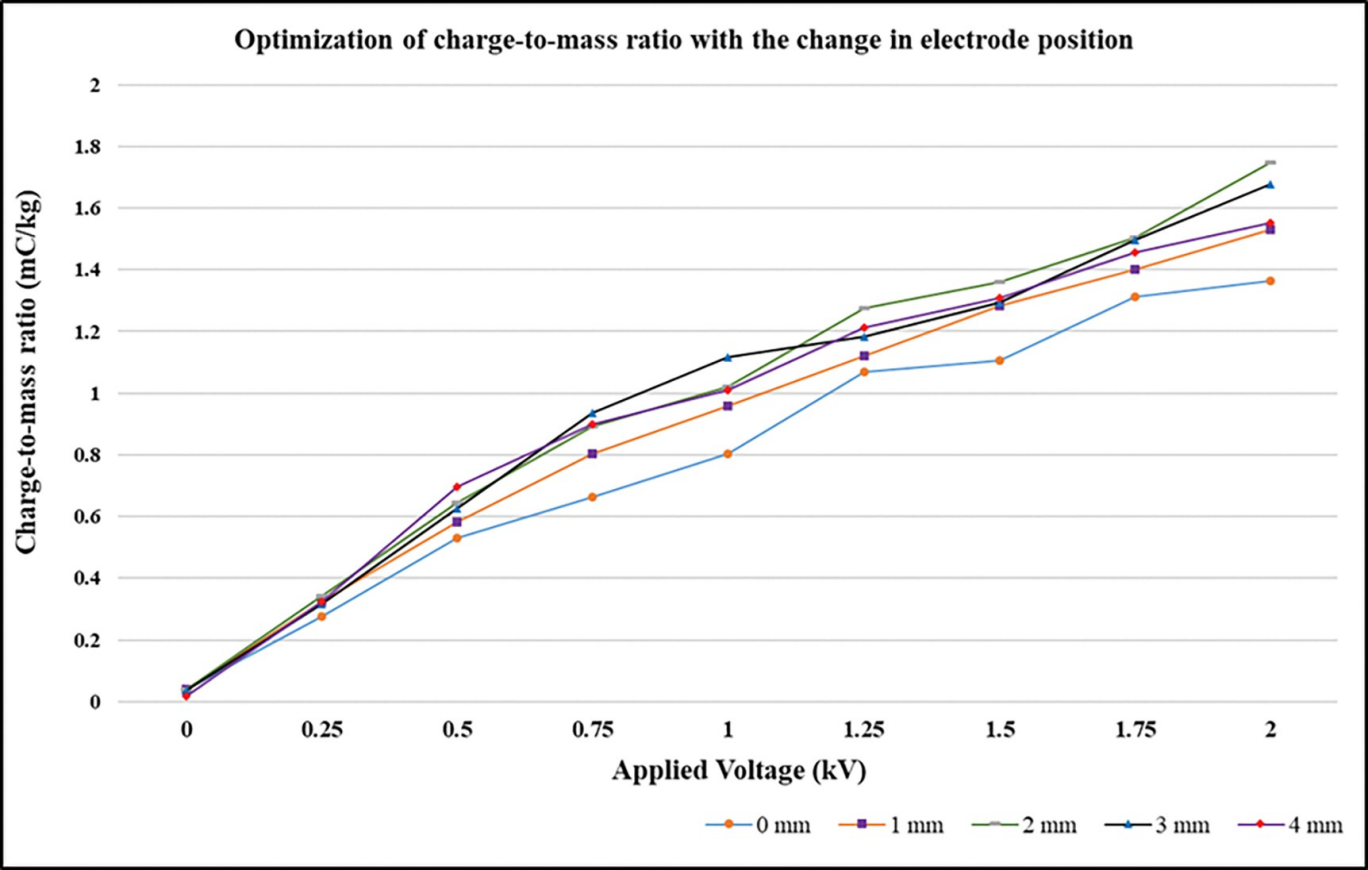
Charge-to-mass ratio variation with the variation of electrode position from the nozzle tip at various applied voltage.

As the distance between the nozzle tip and electrode is increased, the spray current tend to decrease because of space charge effect dominance. Space charge is the presence of droplets around the induction surface. These droplets have separated from the cone- jet due to turbulence in the spray. The space charge weakens the electric field created around the cone- jet spray, this is caused by the droplets in the vicinity of the induction surface that settles down on it causing wetting of the induction surface giving rise to increase in leakage surface current [54]. In Fig 4, charge-to-mass ratio is seen to be increasing linearly for 2 mm distance and at 3 mm and 4 mm distances from the nozzle tip, the charge-to-mass ratio is comparatively less even for highest applied voltage i.e., 2 kV. Therefore, the optimized charge-to-mass ratio is obtained at distances of 0 mm, 1 mm and 2 mm below the spray nozzle.

### 4.2 Charge-to-mass ratio evaluation of different sized charging electrodes and material diameter

**Fig 5:**
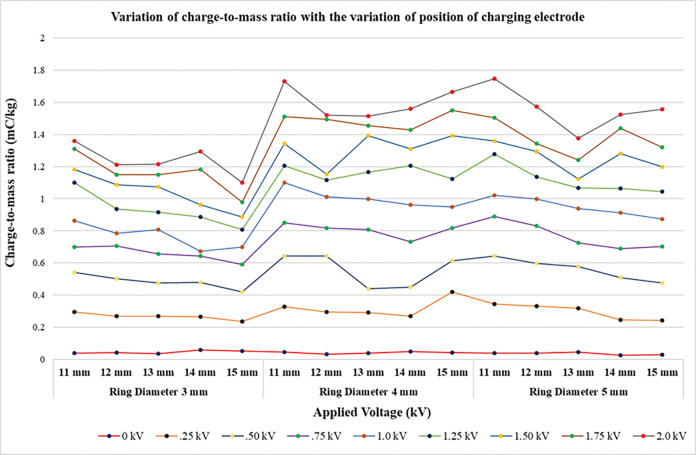
Charge-to-mass ratio variation with varying charging electrode size (inner diameter) and electrode ring material size (outer diameter).

We have chosen a total of 15 electrodes varying in ring diameter and internal diameter. Ring diameter of 3 variations was taken as 3mm, 4mm and 5 mm, each of which had internal diameter as 11mm, 12mm, 13mm, 14mm and 15mm. The electrodes are ring shaped made up of copper material (Fig 4A). As can be seen in Fig 5, the ring diameter do not pose any significant change in the level of the charge-to-mass ratio. But the internal diameter is a major factor contributing to the charge-to-mass ratio. The electrode is the induction surface to which high voltage is applied and the spray is emitted out from the nozzle in a conical shape passing through the electrode (Fig 4B). The spray does not directly come in contact with the induction surface. By the mechanism of induction charging the spray passing through the electrodes acquires charge. The ring with smaller internal diameter induced the maximum charge compared with electrode with larger internal diameter [55]. This caters to the fact that less corona discharge occurs with smaller diameter electrodes. Corona discharge is the phenomenon of ionizing the minute water droplets that exist in the region of electrode. These ionized droplets acquire the charge of opposite nature to that of spray and settles on the surface of electrode, thereby decreasing the overall spray charge.

As seen in graph 5, the charge-to-mass ratio attained by spray with smaller diameter nozzles i.e., 11 mm and 12 mm is greater than 14 mm and 15 mm electrodes.

### 4.3 Charge-to-mass evaluation with respect to varying nozzle diameter and liquid flow rate

**Fig 6:**
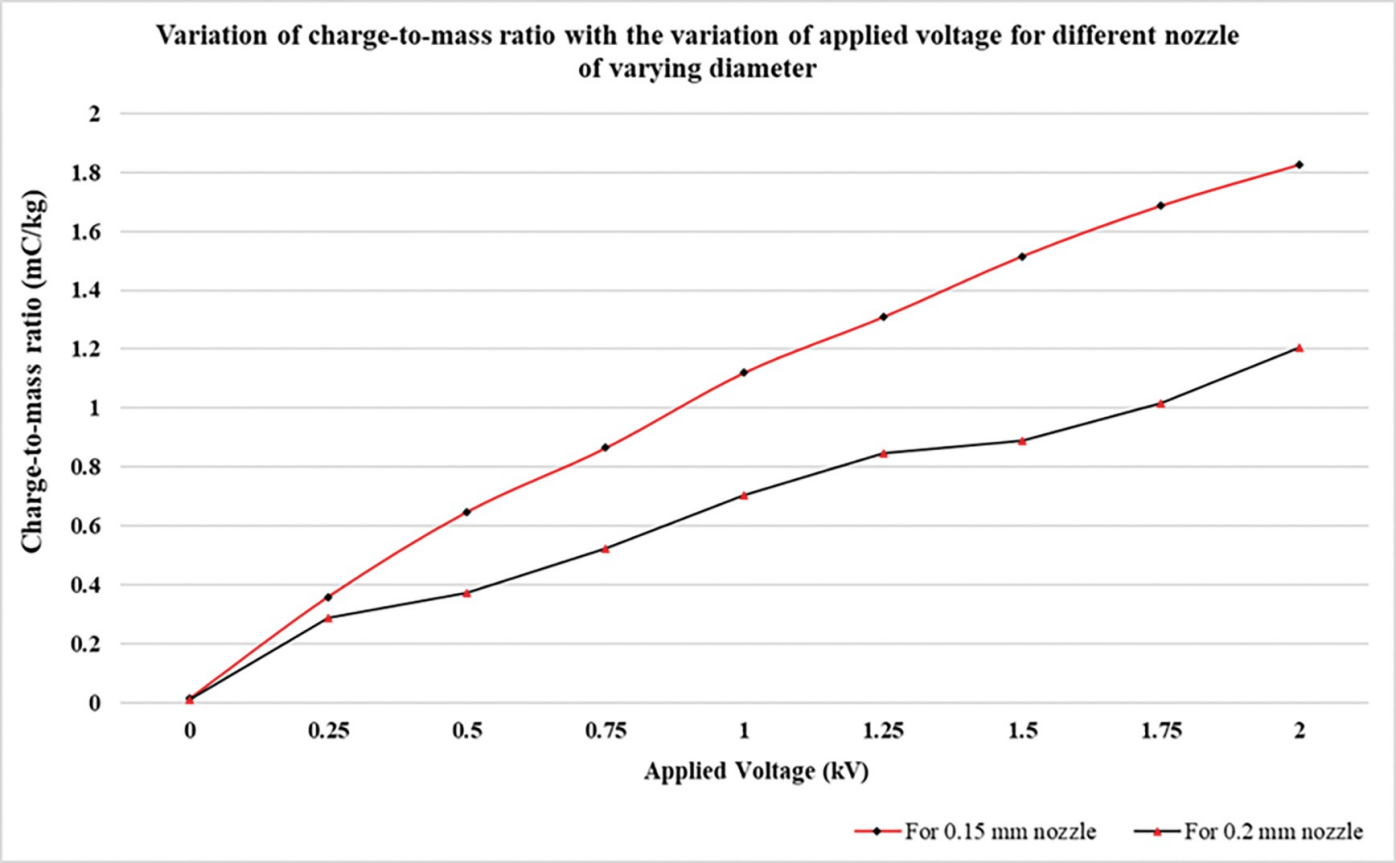
Effect of applied liquid pressure on charge-to-mass ratio and spray current at a fixed applied voltage.

**Fig 7:**
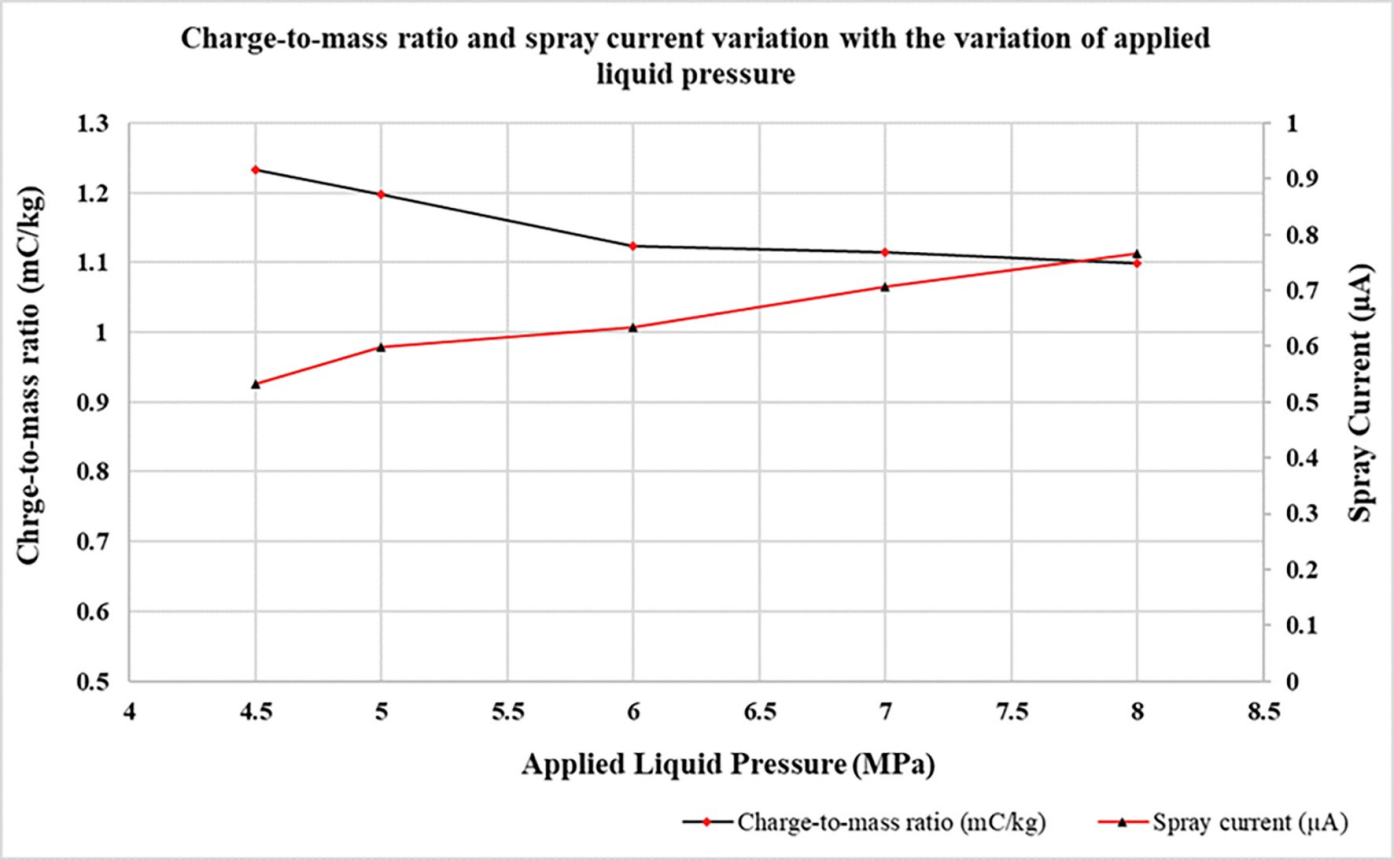
Plot of variation of charge-to-mass ratio with the variation of applied voltage for different nozzle of diameter of 0.15 and 0.2 mm at a fixed liquid pressure.

In electrostatics theory, liquid flow rate and applied pressure are two important parameters that are in direct proportion to each other. As we increase the pressure, correspondingly the liquid flow rate also increases. As can be seen in Fig 6 and Table 2, at higher pressure (and flow rate), the charge-to-mass ratio is smaller, but the spray current is greater. This is because as the spray current goes on increasing, simultaneously flow rate also goes up, thereby decreasing the total charge-to-mass ratio going by the formula. With increasing pressure, droplet size is reduced so in accordance with Rayleigh charge limit which is a function of surface tension and diameter of the droplet, there is small fraction of charge over the liquid droplets [51, 56]. The graph in Fig 7 was obtained with nozzle of 0.15 mm and 0.2 mm diameter and electrode having 4mm ring diameter and 11 mm internal diameter at a constant voltage of 2.0 kV. After varying the nozzle size, we found that charge-to-mass ratio obtained with 0.15mm nozzle was greater that obtained by 0.2 mm nozzle at the same applied voltage as shown in Fig 7. This may be attributed to the fact that nozzle with larger diameter produces more turbulent spray that causes wetting of electrode and weakens the effective electric field around the nozzle and electrode [57]. Hence, the induction charging of droplets decreases resulting in lesser the charge-to-mass ratio.

**Table 2:** Variation of charge-to-mass ratio and spray current with pressure and flow rate.

Pressure (MPa)	Flow rate (ml/min)	Spray current (μA)	Charge-to-mass ratio (mC/kg)
4.5	26	0.532	1.23
5	28	0.599	1.19
6	34	0.634	1.12
7	38	0.706	1.11
8	42	0.766	1.09

### 4.4 Variation of charge-to-mass ratio with varying target distance from the nozzle tip

**Fig 8:**
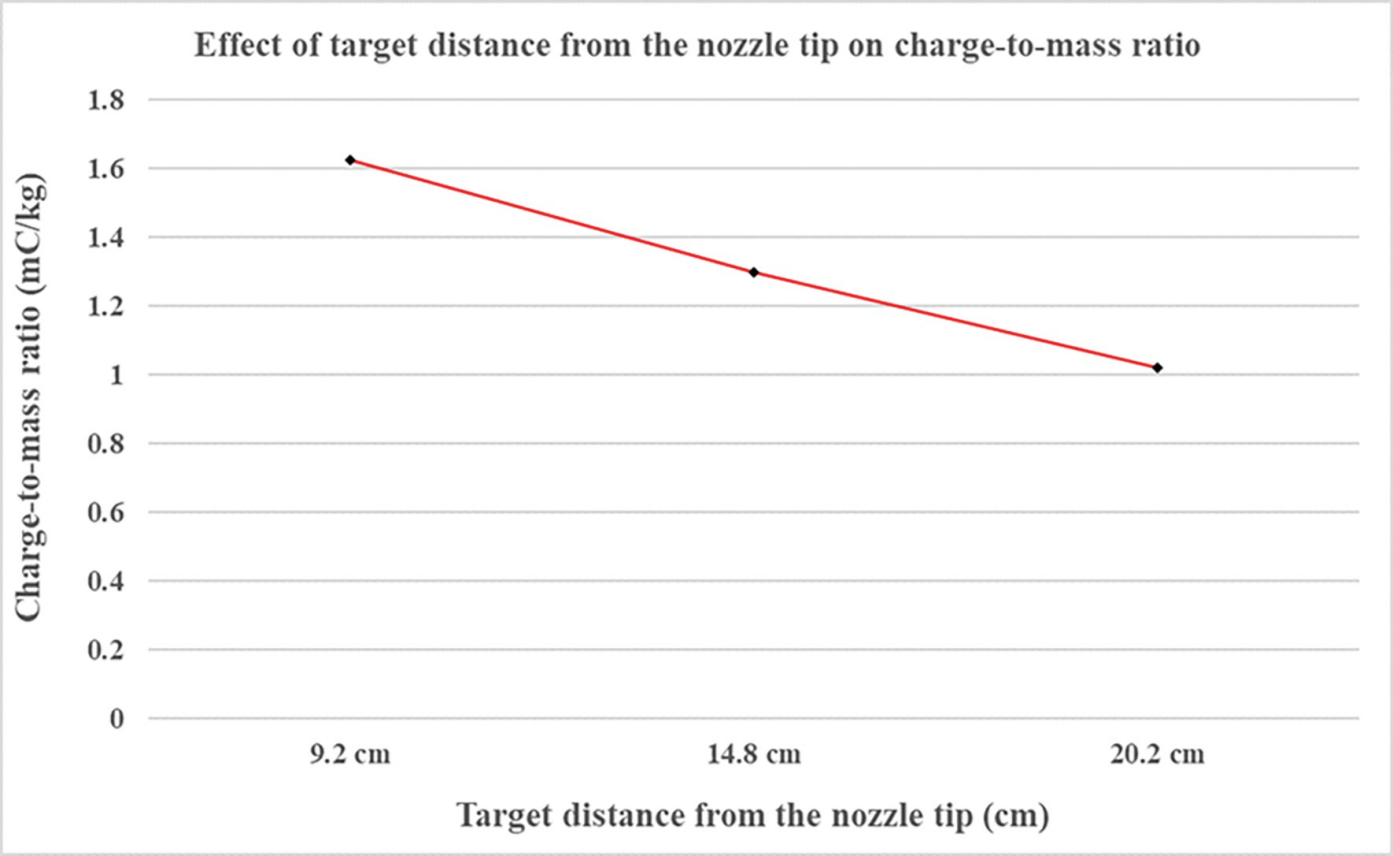
Charge-to-mass ratio variation with the variation of target distance from the nozzle tip.

With increasing target distance, the charge-to-mass ratio tends to decrease as an effect of air resistance faced by droplets that makes them lose electric charge [58]. Also the minute droplets tends to evaporate in the environment and some may deviate from path and settle to ground, thereby decreasing the total effective spray current [54]. Fig 8 shows that as the distance from nozzle to target increased from 9.2 cm to 14.8 cm and further 20.2 cm, there is linear decrease in charge-to-mass ratio.

### 4.5 Chargeability of different disinfectants

Chargeability of the spray liquid droplets is highly influenced by the liquid properties apart from the physical performance parameters [59]. The physical parameters such as, pH, conductivity, liquid density, viscosity and surface tension were measured and mentioned in Table 1. These factors have an effect on the droplet formation process. Factors like surface tension and droplet size determine the maximum charge a droplet can carry also known as ‘Rayleigh limit’. The amount of charge imparted on the droplet depends on the charge transfer time to the droplets and the droplet formation time [60, 61]. The charge transfer time constant *τ* (*s*) is characterized by electrical conductivity *σ* (*mho/m*) and permittivity *Ɛ* (*C*^*2*^*/N*.*m*^*2*^) of the liquid being sprayed as given by Eq 2.


τ=Ɛσ (3)

For effective electrostatic-induction charging, charge transfer time constant should be less than droplet formation time.

**Fig 9:**
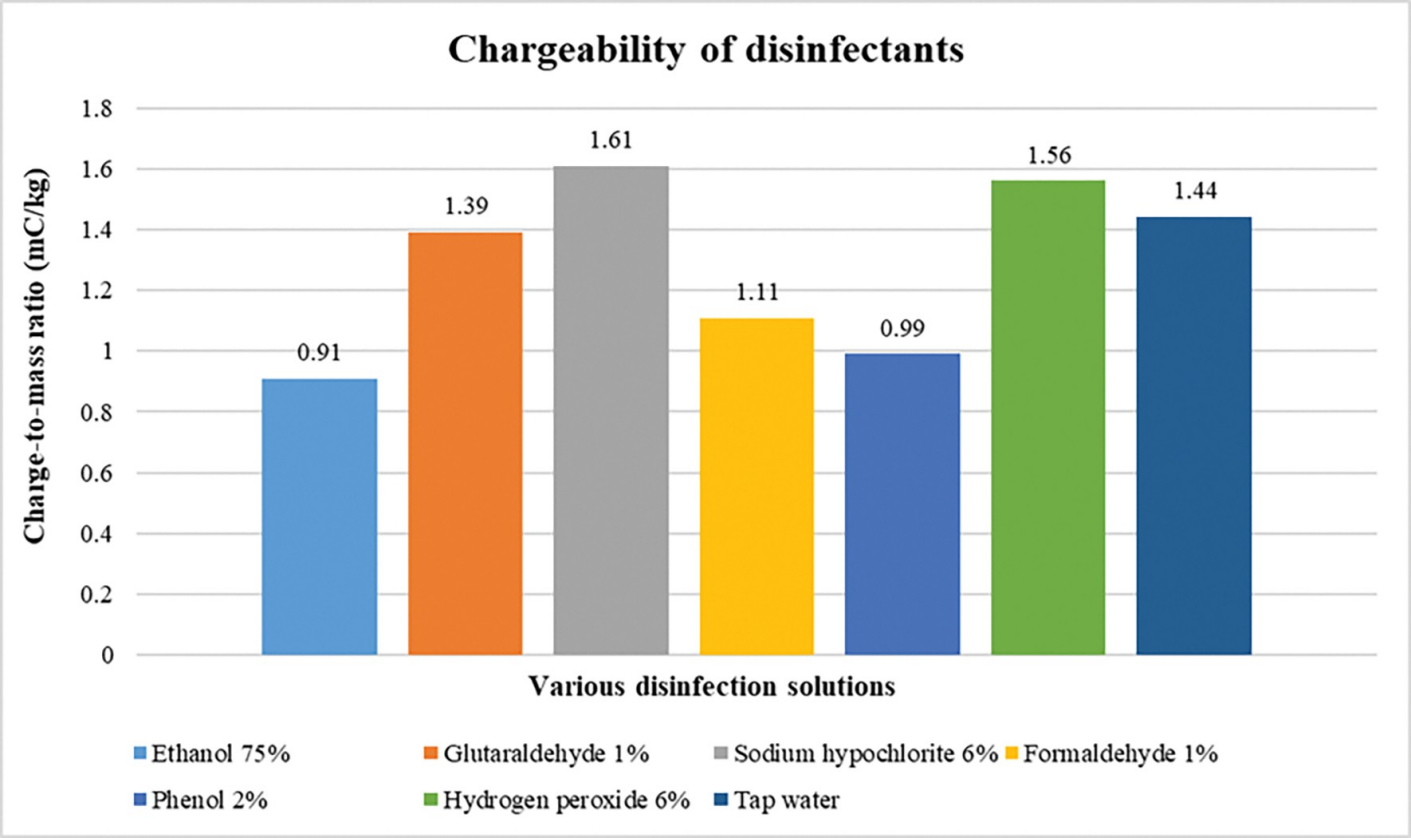
Charge-to-mass ratio of various disinfectant solutions.

In the Fig 9, charge-to-mass ratio of different disinfectant solutions has been plotted at 2 kV voltage with nozzle of 0.15 mm diameter and electrode of 4 mm and 11 mm ring and internal diameter, respectively. The charge-to-mass ratio obtained are seen to be in agreement with the conductivity of respective disinfectant solution.

### 4.6 Measurement of droplet size and its distribution with varying nozzle diameter

**Fig 10:**
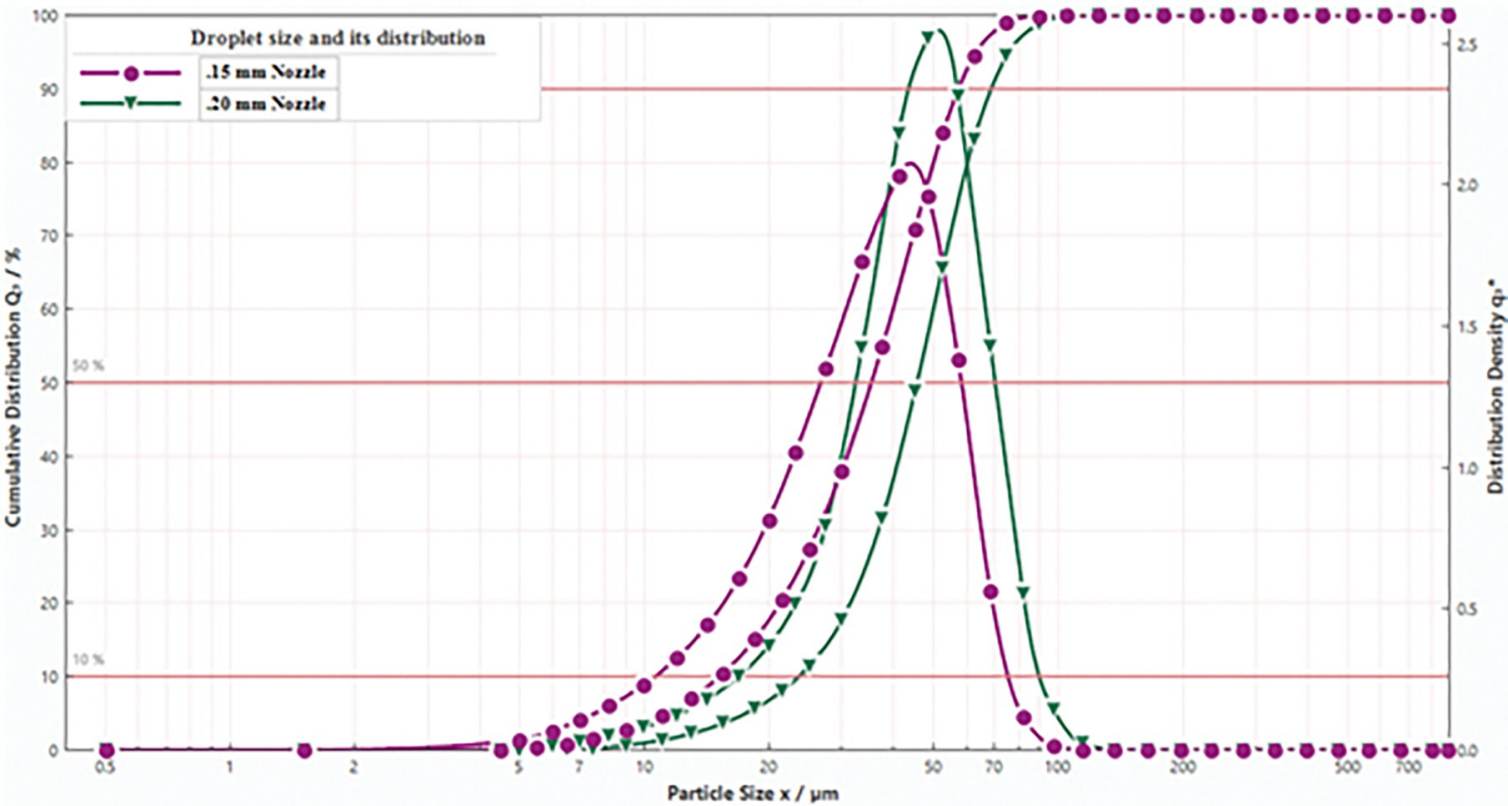
Droplet size and its distribution for 0.15 mm and 0.20 mm nozzles at 5 MPa pressure.

Fig 10 indicates the droplet size and its distribution as obtained with 0.15 mm and 0.2 mm nozzles using Sympatec HELOS/KR- VARIO particle size analyser. The Sauter Mean Diameter (SMD) of 0.15 mm and 0.2 mm nozzles were measured as 36.21 μm and 46.36 μm, respectively. Usually better charge-to-mass ratio is obtained with smaller sized nozzles because small sized nozzle produces spray with narrow cone angle. Narrower is the cone angle lesser is the chance of droplets to hit the induction surface and produce corona discharge. One more reason is the dependency of ‘Rayleigh Limit’ on the droplet size. The Rayleigh Limit creates a balance between the electrostatic force and surface tension forces to maintain the shape and size of droplets [31, 62–64]. The charge-to-mass ratio as calculated using the Rayleigh Limit is expressed as

$$\frac{Qmax}{m}=\frac{\sqrt{2}\times 12\sqrt{Ɛ}\circ \sqrt{ϒ}}{\rho d^{3/2}}$$ (4)

Where, *Qmax* is the Rayleigh charge limit, *m* is the mass of the droplet, *Ɛ*_*0*_ is the free space permittivity, *σ* is the surface tension of droplet (*N/m*), *ρ* is the density of liquid (*kg/m*^*3*^) and *d* is the diameter of droplet (*m*) [33].

Eq 4 supports that smaller is the droplet size, greater is the charge-to-mass ratio achieved by the spray droplets. Experimentally, the charge-to-mass ratio obtained with 0.15 mm nozzle was greater than that obtained with 0.2 mm nozzle as depicted in Fig 6.

**Fig 11:**
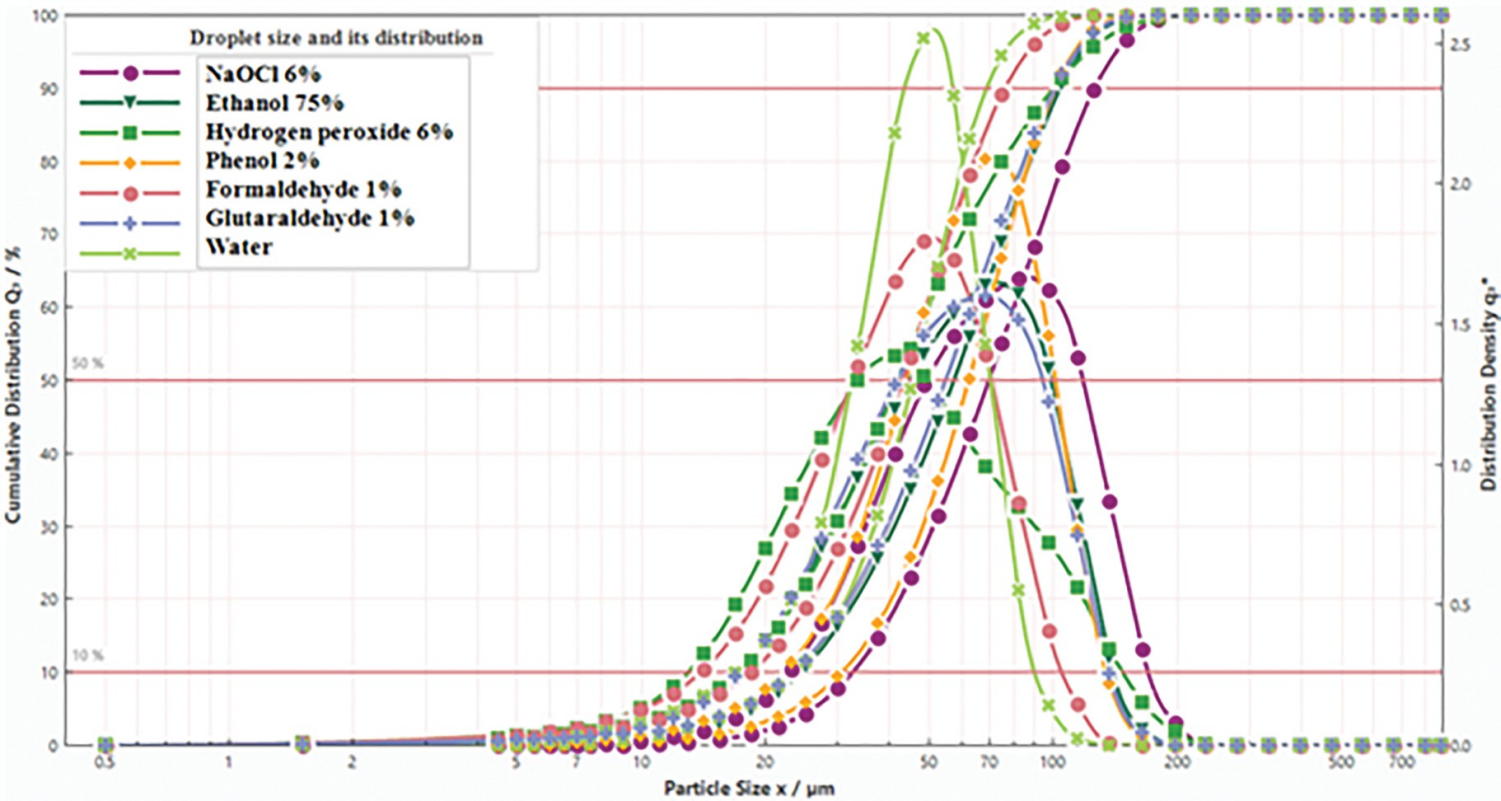
Droplet size and its distribution for various disinfectant solutions.

The droplet size and distribution of various chemical disinfectants is shown in Fig 11 and Table 3.

**Table 3:** Measured charge-to-mass ratio and droplet size of various disinfectant solutions.

Solvents	Charge-to-mass ratio (mC/kg)	Droplet size (Sauter Mean Diameter)
**Ethanol (75%)**	1.261	38.67 μm
**Phenol (2%)**	1.482	44.25 μm
**Hydrogen peroxide (6%)**	1.212	29.88 μm
**Glutaraldehyde (1%)**	1.352	50.78 μm
**Formaldehyde (1%)**	1.298	30.48 μm
**Sodium hypochlorite (6%)**	1.259	57.02 μm
**Tap water**	1.731	27.23 μm

## 5. Conclusion

In this study, the effect of various parameters on the charge-to-mass ratio have been investigated. More is the charge-to-mass ratio, better is the deposition of spray over the target surface. From the experiments it was observed that the maximum the charge-to-mass ratio of 1.82 mC/kg was obtained at 2.0 kV at an applied pressure of 5 MPa and liquid flow rate of 28 ml/min with the nozzle size of 0.15 mm. Using the same nozzle if we increase the pressure and simultaneously the flow rate, the charge-to-mass ratio was seen to be decreasing, although minutely. This was because as the flow rate increased with applied pressure, wider cone-angle spray was produced that wet the induction surface leading to corona discharge. Ring electrode with smaller induction surface charged the spray droplets better at a smaller gap between nozzle outlet and ring electrode. As this gap increased, the charge-to-mass ratio decreased due to leakage current.

Typically, smaller sized droplets hold a better charge for the charge-to-mass ratio being a function of droplet size as per Eq 4. Chargeability of disinfectant solutions were seen to be moderate in compliance with the conductivity. At a certain value of the conductivity, the chargeability is optimum and then decreases in both the direction and forms i.e. by decreasing or increasing from the optimum value of conductivity.

## Supporting information

S1 Data Variation of CMR Vs liquid pressure (flowrate). (XLSX)

S2 Data Calculations and graphs. (XLSX)
